# Performance Improvement of a Vehicle Equipped with Active Aerodynamic Surfaces Using Anti-Jerk Preview Control Strategy

**DOI:** 10.3390/s22208057

**Published:** 2022-10-21

**Authors:** Ejaz Ahmad, Iljoong Youn

**Affiliations:** Department of Mechanical and Aerospace Engineering, Gyeongsang National University, ReCAPT, Jinju 52828, Korea

**Keywords:** anti-jerk control, preview control, half-car model, bump input, aerodynamics, asphalt road, road holding

## Abstract

This paper presents a formulation of a preview optimal control strategy for a half-car model equipped with active aerodynamic surfaces. The designed control strategy consists of two parts: a feed-forward controller to deal with the future road disturbances and a feedback controller to deal with tracking error. An anti-jerk functionality is employed in the design of preview control strategy that can reliably reduce the jerk of control inputs to improve the performance of active aerodynamic surfaces and reduce vehicle body jerk to enhance the ride comfort without degrading road holding capability. The proposed control scheme determines proactive control action against oncoming potential road disturbances to mitigate the effect of deterministically known road disturbances. The performance of proposed anti-jerk optimal control strategy is compared with that of optimal control without considering jerk. Simulation results considering frequency and time domain characteristics are carried out using MATLAB to demonstrate the effectiveness of the proposed scheme. The frequency domain characteristics are discussed only for the roll inputs, while time domain characteristics are discussed for the corresponding ground velocity inputs of bump and asphalt road, respectively. The results show that using anti-jerk optimal preview control strategy improves the performance of vehicle dynamics by reducing jerk of aerodynamic surfaces and vehicle body jerk simultaneously.

## 1. Introduction

During the past few decades, the significant advancement in the design of automobiles employing active suspension components has resulted in an improvement in ride comfort and road holding. Even though the ride comfort in the vehicle ride has vastly improved, driving on uneven roads is still a serious concern for automotive engineers [1]. The ride comfort and road holding capability are the two essential aspects of the vehicle 25 suspension system, which have received the attention of many researchers in this field [2,3,4,5,6]. In ride comfort, the vibrations of the vehicle body are isolated by reducing the effect of noise and harshness associated with road disturbances. Road holding involves reducing variations in typical wheel load on the tire to optimize the tire grip on the road [7,8]. Both these aspects are contradictory, and it is challenging for an automobile designer to strike a balance between them within the constraints of vehicle geometry. Hence, there is a need for an effective control systems framework that can conveniently address these two aspects so that ride quality can be enhanced.

Control system theory plays an essential role in increasing vehicle performance by adjusting the power input required by the actuators. Researchers in the automobile industry have utilized various dynamical models with different control strategies to evaluate vehicle performance in terms of ride comfort and road holding capabilities. In [9,10,11,12,13], the authors used a sophisticated two Degree Of Freedom (DOF) quarter vehicle model to improve ride comfort and road holding and utilized Linear Quadratic Regulator (LQR) and fuzzy logic control approaches. Similarly, in [14,15,16], a more complicated and ubiquitous four DOF half-car model that can be treated as a longitudinal or lateral vehicle model with an extra two DOF of roll and heave motion, crucial for ride comfort and road holding capability. In [17], a nonlinear backstepping-based active suspension technique was used to minimize the trade-off between ride comfort and road holding capabilities. Artificial Intelligence (AI)-based control algorithms were formulated to enhance the vehicle ride quality using active suspension system [18,19,20,21]. Recently, in [22], a network-based intermittent and quantized data transmission architecture for networked active suspension of a half in-wheel motor electric car is presented to optimize vehicle performance. These control techniques are primarily based on semi-active or fully active suspension system control procedures. Moreover, in these conventional suspension-based strategies, the actuators are installed between sprung mass and unsprung mass, which is the main reason to cause a trade-off between ride comfort and road holding. Hence, the prior research is based on conventional active and semi-active suspension-based control schemes, but recently the research on active aerodynamic-based control schemes have achieved extensive momentum, which provide the necessary control forces directly to the sprung mass to overcome the trade-off between the ride comfort and road holding.

With the advent of high-speed vehicles, aerodynamics has become increasingly important in terms of improving ride comfort and ride holding capability. The vehicle outfitted with Active Aerodynamic Surfaces (AAS) significantly increases negative lift force with vehicle speed, improving ride comfort and road holding capabilities. However, this complicates the dynamics of the vehicle models, necessitating the use of a sophisticated control strategy to handle such complicated dynamics. Various control approaches for the control of vehicles equipped with AAS exist in the literature, resulting in considerable improvements in ride comfort and road holding capabilities. The preliminary research work for the control of vehicles equipped with aerodynamic surfaces has been reported in [23,24,25] utilizing several Active Aerodynamics Control (AAC) techniques to enhance the ride comfort. Doniselli et al. investigated the effects of aerodynamics on the ride quality of a high-speed vehicle on a randomly contoured road [26]. The vehicle performance in terms of ride comfort and road holding is analyzed in [27] using a model-based controller for a classical quarter car model. In [28,29,30,31], the authors designed various control schemes for the sports cars equipped with active aerodynamic surfaces and ensured improvement in the ride comfort and road holding capability. In our previous work [32] we used an AAC strategy to improve the ride comfort and road holding capability of the vehicle. In aerodynamics-based vehicles, the essential issue is the realistic motion of aerodynamic surfaces fitted on sprung mass. Because of the high speed, the abrupt movement of aerodynamic surfaces can cause substantial vehicle body jerk and acceleration, which can have a negative impact on passenger ride comfort. A suitable solution to mitigate the negative impact and ensure the ride comfort and road holding capability is referred to as anti-jerk-based control scheme. The anti-jerk control approach is commonly used in commercial vehicles to reduce the effect of acceleration oscillations that occur during longitudinal, and lateral motion of the vehicle body [33]. In [34], an anti-jerk control technique based on deep reinforcement learning is applied to analyze the relationship between collision avoidance for safety and jerk reduction. An explicit nonlinear model predictive based anti-jerk controller is designed in [35,36] to improve ride comfort. To accommodate for model uncertainty, [37] implements a μ-synthesis robust anti-jerk controller for electric vehicles. However, these algorithms were developed for longitudinal jerk of the vehicle body, and vertical vehicle body jerk was not taken into account in the design problem. For a half-car model the vehicle body jerk includes heaving and angular jerks.

Preview control scheme is considered to be an impressive control strategy for the vehicle performance against future information about road disturbances. These disturbances can be detected using a look-ahead sensors, installed in the front of the vehicle. The look-ahead sensor is used by many researchers [38,39,40] to obtain the preview information about future road irregularities. In our previous work, a look-ahead sensor [41] is used for an active suspension system a 3 DOF full tracked vehicle. The preview control has a vast applications in vehicle systems as well as other engineering fields, see a review by [42]. In [43], the authors used preview control for path tracking and future landing maneuvers. A preview control designed in [44,45] is used to improve the ride comfort and road holding of vehicle during cornering. In [46] a fuzzy preview control strategy is developed to improve suspension performance of a half-car model. Based on the applications of preview controller, the main objective of this paper is to investigate the role of anti-jerk optimal preview controller in passenger ride comfort and vehicle handling.

Motivated by these observations, in this manuscript we consider a four-degree-of-freedom half-car model equipped with active aerodynamic surfaces that accounts vertical acceleration of the vehicle body. The aim is to formulate an anti-jerk preview control (AJPC) strategy based on the feedback and feed-forward control that can reliably reduce the jerk of AAS to improve its performance, and reduce the vehicle body jerk and other outputs to improve the ride comfort while maintaining the road holding capabilities. The distinguishing features of this work are given as follows.

- A comprehensive case study for the control of half-car model equipped with active aerodynamics surfaces is presented.
- We consider an anticipation of future values of deterministically known road disturbances in the design problem.
- To reduce the effect of jerk related to the ride comfort of a vehicle, a weighted norm of jerk control input is included in the pre-specified performance index. The minimization of the performance index accounts for improvisation of ride comfort and road holding capability.
- The feed-forward controller is designed based on the formulation for the preview control subjected to oncoming measured road disturbances.
- Simulations were carried using MATLAB that demonstrates the effectiveness of the proposed scheme in terms of minimizing control jerk, and assuring ride comfort and road holding capabilities. It was also demonstrated that the designed control algorithm has a proclivity to anticipate future measured disturbances and to take remedial action accordingly ahead of the occurrence of disturbances.

The rest of paper is organized as follows. Section 2 presents mathematical modeling of a half-car with aerodynamic surfaces. Section 3 provides the problem formulation. Section 4 presents the formulation of anti-jerk preview control strategy for the systems having measured disturbances. Simulation results and discussions are provided in Section 5. Finally, the concluding remarks and future directions are drawn in Section 6.

## 2. Mathematical Modeling of Vehicle

**Figure 1:**
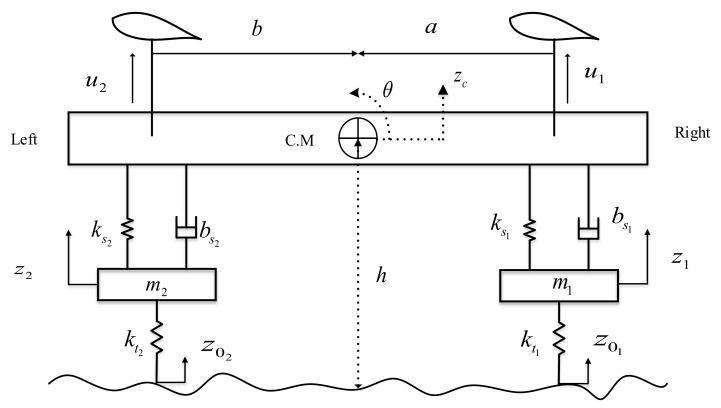
Schematic diagram for a half-car model equipped with aerodynamic surfaces.

In this paper, a four degree-of-freedom half-car model with active aerodynamic surfaces is used to investigate its performance in the presence of detected oncoming road disturbances. Figure 1 depicts a schematic representation of the proposed model, which features active aerodynamic surfaces. The proposed model comprised of one sprung and two unsprung masses. The sprung mass can experience a heave and roll motion, while the unsprung masses are free to bounce vertically. AAS are mounted at right and left position of sprung-mass system to provide the necessary control forces $u_{1}$ and $u_{2}$. $z_{c}$ represents the vertical displacement of sprung mass, while $z_{1}$ and $z_{2}$ represents the vertical displacements of unsprung masses, θ is the roll angle and C.M is known as the center of mass of the vehicle body. *a* and *b* are the distances between the mounting points of suspension system and C.M. $z_{01}$ and $z_{02}$ represent the road disturbances given to right and left sides respectively. Table 1 presents the prominent vehicle parameters and their values considered in this paper.

The vehicle model is derived using Newtonian’s second law, where Equation (1) represents the equation of motion for the sprung mass acceleration as:

(1) $$\begin{array}{cc} M{\ddot{z}}_{c} & =f_{r}+f_{l}+u_{1}+u_{2} \end{array}$$

where *M* is the sprung mass, $f_{r}$ and $f_{l}$ are forces generated due to the aerodynamic and suspension forces acting at mounting points as given in (2) and (3).

(2) $$\begin{array}{cc} f_{r} & =k_{s_{1}}\left(z_{1}-z_{c}-a\theta \right)+b_{s_{1}}\left({\dot{z}}_{1}-\dot{z_{c}}-a\dot{\theta }\right) \end{array}$$

(3) $$\begin{array}{cc} f_{l} & =k_{s_{2}}\left(z_{2}-z_{c}+b\theta \right)+b_{s_{2}}\left({\dot{z}}_{2}-\dot{z_{c}}+b\dot{\theta }\right) \end{array}$$

where $k_{s_{1}}$ and $k_{s_{2}}$ are spring stiffness coefficients, $b_{s_{1}}$ and $b_{s_{2}}$ are called damping coefficients at right and left sides. Equation (4) represent the roll equation of motion.

(4) $$\begin{array}{cc} I\ddot{\theta } & =\left(f_{r}+u_{1}\right)a-\left(f_{l}+u_{2}\right)b \end{array}$$

where *I* is the moment of inertia of the vehicle. Equations (5) and (6) represent the unsprung mass equations for the right and left sides.

(5) $$\begin{array}{cc} m_{1}{\ddot{z}}_{1} & =-k_{t_{1}}\left(z_{1}-z_{0_{1}}\right)-f_{r} \end{array}$$

(6) $$\begin{array}{cc} m_{2}{\ddot{z}}_{2} & =-k_{t_{2}}\left(z_{2}-z_{0_{2}}\right)-f_{l} \end{array}$$

where $m_{1}$ and $m_{2}$ represents the unsprung masses, $k_{t_{1}}$ and $k_{t_{2}}$ are the tire stiffnesses. As discussed, in this work, we are designing an anti-jerk control strategy with preview information. Therefore, the jerk control input can only be introduced to the system by differentiating (1) and (4) to obtain (7) and (8) respectively.

(7) $$\begin{array}{cc} M{\dddot{z}}_{c} & ={\dot{f}}_{r}+{\dot{f}}_{l}+{\dot{u}}_{1}+{\dot{u}}_{2} \end{array}$$

(8) $$\begin{array}{cc} I\dddot{\theta } & =\left({\dot{f}}_{r}+{\dot{u}}_{1}\right)a-\left({\dot{f}}_{l}+{\dot{u}}_{2}\right)b \end{array}$$

### 2.1. Aerodynamic Forces

The main goal of this research is to adopt an anti-jerk optimum control method based on active aerodynamics to increase AAS performance and ride quality. The installation of AAS on sprung mass produces the required control forces to affect the vertical motion of the vehicle body. These forces can modify the sprung mass system’s vertical load, which in turn affects the acceleration of pitching and heaving, tyre deflection, and suspension deflection. The aerodynamic surfaces forces, $u_{1}$ and $u_{2}$, are given as follows:

(9) $$\begin{array}{c} u_{1,2}=\frac{1}{2}\rho v^{2}SC_{lift}\left(\alpha \right) \end{array}$$

where ρ is the air density, *v* is the speed of the vehicle, *S* is the surface area of the airfoil, and $C_{lift}$ is the lift coefficient, which depends on the angle of attack (α) and the surface area of the airfoil.

#### Road Excitation Model

**Figure 2:**
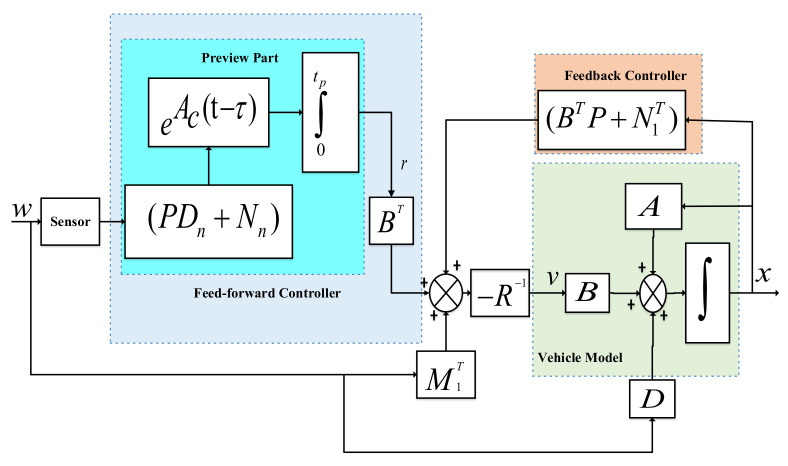
Block diagram schematic comprised of feed-forward to detect the future road disturbance and feedback controller responsible for tracking error.

This, study consists of the measurement of road profile from the sensor data, and to utilize it to design an active aerodynamic based preview control strategy. In order to evaluate the performance of the proposed controller with respect to the vehicle suspension performance characteristics, two types of disturbance inputs, a bump velocity input an asphalt road disturbances given to the right are considered. As shown in Figure 2, the sensor detect these disturbances ahead of the vehicle. The bump input given in Equation (11) can be assumed as shocks.

(10) $$\begin{array}{c} z_{0_{1}}=\left\{\begin{array}{cc} c\left[1-\cos40\pi (t-0.5)\right] & \;\mathrm{for}t\in \left[0.5,0.55\right]\\ 0 & \;\;\mathrm{otherwise} \end{array}\right. \end{array}$$

The following equation represent the corresponding ground velocity input.

(11) $$\begin{array}{c} w_{1}=\left\{\begin{array}{cc} 40c\pi \sin40\pi (t-0.5) & \;\mathrm{for}t\in \left[0.5,0.55\right]\\ 0 & \;\;\mathrm{otherwise} \end{array}\right. \end{array}$$

where $w_{1}={\dot{z}}_{0_{1}}$ and $w_{2}={\dot{z}}_{0_{2}}$, 2c=0.1 m is called the height of the bump input. $z_{0_{2}}$ is zero in case of bump input to left side. The second type of disturbance is an asphalt road disturbance w(t), which is assumed as white noise with zero mean value (w(τ),$\tau \in [t,t+t_{p}])$, where the preview time $t_{p}$ is deterministically known. As given in our previous work [47], the co-variance of w(t) is utilized to construct the road input for the simulation.

(12) $$E\left[w_{1}\left(t_{1}\right)w_{1}\left(t_{2}\right)\right]=2\pi aV\delta \left(t_{1}-t_{2}\right)$$

where a=0.15 m represents the road’s roughness for the asphalt road, and *V* represents the vehicle speed of 20 m/s.

The corresponding performance index that need to be minimized by suitable control law, and can be expressed in terms of heaving acceleration, roll acceleration, suspension deflection, roll angle, tyre deflection and jerk control input is given by

(13) $$\begin{array}{cc} J=\underset{\tau \to \infty }{lim}\;\frac{1}{2T}\int_{0}^{T} & \left[\rho_{3}{\left(z_{c}+a\theta -z_{1}\right)}^{2}+\rho_{3}{\left(z_{c}-b\theta -z_{2}\right)}^{2}+\rho_{5}{\left(z_{1}-z_{01}\right)}^{2}+\rho_{5}{\left(z_{2}-z_{02}\right)}^{2}\right.\\ & \left.+\rho_{2}{\ddot{\theta }}^{2}+\rho_{4}\theta^{2}+\rho_{1}{\ddot{z}}_{c}^{2}+\rho_{7}\left({\dot{u}}_{1}^{2}+{\dot{u}}_{2}^{2}\right)\right]d\tau \end{array}$$

The weighting constants $\rho_{1}$, $\rho_{2}$, $\rho_{3}$, $\rho_{4}$, $\rho_{5}$, $\rho_{7}$ is multiplied to the rms of heaving acceleration, roll acceleration, suspension deflection, roll angle, tyre deflection and jerk control input, reflecting the preferences of the designer. These weights determine appropriate balance between numerous components of the optimized criterion.

## 3. Problem Formulation

### 3.1. System Description

Consider a half car whose dynamics can be represented by the following continuous-time state-space model.

(14) $$\begin{array}{cc} \dot{x}\left(t\right) & =Ax(t)+Bv(t)+Dw(t) \end{array}$$

where $x\left(t\right)\in R^{n}$ is the system’s state vector (n = 12), $v\left(t\right)=\dot{u}\in R^{m}$ is the jerk control input (m = 2), and $w\left(t\right)\in R^{q}$ is called deterministically known road disturbance (q = 2).

$$\begin{array}{cc} x & =\left[z_{c}\;{\dot{z}}_{c}\;{\ddot{z}}_{c}\;\theta \;\dot{\theta }\;\ddot{\theta }\;z_{1}-z_{0_{1}}\;{\dot{z}}_{1}\;z_{2}-z_{0_{2}}\;{\dot{z}}_{2}\;z_{1}\;z_{2}\right] \end{array}$$

$$\dot{u}={\left[\begin{array}{cc} {\dot{u}}_{1} & {\dot{u}}_{2} \end{array}\right]}^{T},w={\left[\begin{array}{cc} w_{1} & w_{2} \end{array}\right]}^{T}$$

A, B, and D are constant matrices with appropriate dimensions. The non-zero elements of these matrices are given in Appendix A. The following assumptions are considered for the addressed system.

**Assumption** **A1.** *It is assumed that all the states of the system under consideration are measurable and available for feedback.*

**Assumption** **A2.** *The tire damping coefficient can be ignored because it is much smaller than tire stiffness, and it is assumed that the wheel is always in contact with the ground.*

**Assumption** **A3.** *The drag forces generated by active aerodynamics surfaces and vehicle’s body are ignored, and the actual dynamics of the AAS are beyond the scope of this study. Therefore, only the control jerk caused by the AAS is taken into account.*

### 3.2. Controller

The control law v(t) in Equation (14) can be computed by the following equation.

(15) $$\begin{array}{c} v\left(t\right)=f\left(x\left(\tau \right),w\left(\sigma \right),\;\;t_{o}\leq \tau \leq t,\;\;t_{o}\leq \sigma \leq t+t_{p}\right) \end{array}$$

where $t_{o}$ is the initial time and $w\left(\emptyset \right),\tau \in \left[t,t+t_{p}\right]$ denotes deterministically known disturbance that are available to incorporate in the design problem. The required control law presented in (15) can be obtained by minimizing the following performance index.

(16) $$\begin{array}{cc} J\left(x,v,w\right)=\underset{\tau \to \infty }{lim}\frac{1}{2T}\int_{0}^{T} & {\left[\begin{array}{c} x(t)\\ v(t)\\ w(t) \end{array}\right]}_{\tilde{Q}}^{2}d\tau \end{array}$$

where

$$\begin{array}{c} \tilde{Q}=\left[\begin{array}{ccc} Q & 2N_{1} & 2N_{2}\\ 0 & R & 0\\ 0 & 2M_{1} & M_{2} \end{array}\right] \end{array}$$

and $Q=\left[q_{ij}\right]$, $M_{2}=\left[{m_{2}}_{ij}\right]$, $R=[r_{ij}]>0$ and $N_{1}=\left[{n_{1}}_{ij}\right]$, $N_{2}=\left[{n_{2}}_{ij}\right]$$M_{1}={m_{1}}_{ij}$ are weight matrices defined in Appendix A.

Where the entries $a_{{3,6}_{i}}$ are the corresponding entries of state matrix A in third and sixth rows, and *i*th columns. $b_{{3,6}_{j}}$ represents the entries of input matrix B in the third and sixth row, and *j*th columns. $d_{{7,9}_{i}}$ are the corresponding entries of disturbance matrix D in the seventh and ninth row, and *i*th columns. $\psi_{ij}$ represents the diagonal entries of weight matrix Q are given in Appendix A.

For the stat-space model in (14), the main objective is to design an anti-jerk based optimal control strategy defined in (15) that tends to reduce the effect of measured disturbances. This will manage the control jerk of AAS to reduce the vehicle body jerk to enhance ride comfort. Figure 2 shows block diagram that outlines the concept of the proposed approach of using measured future road disturbances in order to improve vehicle ride quality. The control strategy depicted in Figure 2 comprised of two parts, state feedback part $-R^{-1}\left(N_{1}^{T}+B^{T}P\right)x\left(t\right)$, which is the same as a classical Linear Quadratic problem, and a feed-forward part $-R^{-1}\left(M_{1}^{T}w+B^{T}r\right)$ that uses all available future information about the road disturbance.The preview part can be viewed as a generalized feed-forward term which provides an anticipating action based on the predicted system response to the road disturbance detected by the sensor ahead of the vehicle. Actually, the preview control scheme is constructed with the presumption that a look-ahead sensor can accurately gather a preview profile before a car encounters road disturbances.

## 4. Optimal Based Anti-Jerk Preview Control

In this section, an anti-jerk optimal preview controller is designed, which helps to reduce the rms of control jerk, vehicle body jerk and acceleration, roll acceleration, tire deflection, roll angle, and suspension deflection to the control jerk of AAS and enhance ride comfort and maintain road holding capability. To obtain the jerk control input defined in (15), the corresponding Hamiltonian function in (17), is used to determine the proposed control scheme represented by Equation (18)

(17) $$\begin{array}{cc} H(x,v,w) & =\frac{1}{2}\left({x^{T}\left(t\right)}_{Q}^{2}+2x^{T}\left(t\right)N_{1}v\left(t\right)+\lambda \dot{x}\left(t\right)+2x^{T}\left(t\right)N_{2}w\left(t\right)+{v(t)}_{R}^{2}\right.\\ & \left.+2w{\left(t\right)}^{T}M_{1}v\left(t\right)+{w(t)}_{M_{2}}^{2}\right) \end{array}$$

The proposed controller is determined by using the gradient of Equation (17) with respect to the control input v(t), i.e., $\frac{\partial H(x,v,w)}{\partial v}=0$. This yields

(18) $$\begin{array}{cc} v(t) & =-R^{-1}\left[x^{T}\left(t\right)N_{1}^{T}+M_{1}^{T}w\left(t\right)+B^{T}\lambda \left(t\right)\right] \end{array}$$

Now substituting (18) into (14), the resultant state-space equation can be written as

(19) $$\begin{array}{cc} \dot{x}\left(t\right) & =A_{n}x\left(t\right)-BR^{-1}B^{T}\lambda \left(t\right)+D_{n}w\left(t\right) \end{array}$$

where λ is known as Lagrange multiplier interpreted as co-state variable and formulated as the minimization of the Hamiltonian function. Hence, computing gradient of (17) with respect to x(t) i.e., $\frac{\partial H}{\partial x}=-\lambda$, and then performing simple manipulation gives

(20) $$\begin{array}{cc} \dot{\lambda }\left(t\right) & =Q_{n}x^{T}\left(t\right)+N_{n}w\left(t\right)-A_{n}^{T}\lambda \left(t\right) \end{array}$$

where $A_{n}=A-BR^{-1}N_{1}^{T}$, $Q_{n}=Q-N_{1}R^{-1}N_{1}^{T}\geq 0,N_{n}=N_{2}-N_{1}R^{-1}M_{1}^{T}$. The Lagrange multipliers λ is given by

(21) $$\begin{array}{cc} \lambda (t) & =Px(t)+r(t) \end{array}$$

Now differentiating Equation (21) and substituting Equation (19) gives

(22) $$\begin{array}{cc} \dot{\lambda }\left(t\right) & =\left(\dot{P}+A_{n}^{T}P+PA_{n}-{\mathrm{PBR}}^{-1}B^{T}P\right)x\left(t\right)-{\mathrm{PBR}}^{-1}B^{T}r\left(t\right)+{\mathrm{PD}}_{n}w\left(t\right)+\dot{r}\left(t\right) \end{array}$$

To eliminate λ, comparing Equation (20) with (22) gives

(23) $$\begin{array}{cc} \; & \left(\dot{P}-{\mathrm{PA}}_{n}-A_{n}^{T}P+{\mathrm{PBR}}^{-1}B^{T}P-Q_{n}\right)x\left(t\right)=-\dot{r}\left(t\right)\\ & +\left({\mathrm{PBR}}^{-1}B^{T}-A_{n}^{T}\right)r\left(t\right)-\left.({\mathrm{PD}}_{n}+N_{1}\right)w\left(t\right) \end{array}$$

Equation (23) holds if and only if

(24) $$\begin{array}{cc} {\mathrm{PA}}_{n}+A_{n}^{T}P-{\mathrm{PBR}}^{-1}B^{T}P+Q_{n} & =0 \end{array}$$

and

(25) $$\begin{array}{cc} \dot{r}\left(t\right) & =\left({\mathrm{PBR}}^{-1}B^{T}-A_{n}^{T}\right)r\left(t\right)-\left({\mathrm{PD}}_{n}+N_{n}\right)w\left(t\right) \end{array}$$

The solution is closely related to the classical linear optimal quadratic regulator problem except the presence of vector r(t) in the controller that uses all the future information about the road disturbance.

If the pair (A,B) is stabilizing and (A,Q) are detectable, then the following anti-jerk controller is obtained by putting Equation (21) in (18) yields

(26) $$\begin{array}{cc} v(t) & =-R^{-1}\left[\left(N_{1}^{T}+B^{T}P\right)x\left(t\right)+M_{1}^{T}w\left(t\right)+B^{T}r\left(t\right)\right] \end{array}$$

Substituting Equation (26) in (14), the close loop equation become:

(27) $$\begin{array}{cc} \dot{x} & =\mathrm{Ax}-B\left[R^{-1}\left(\left(N_{1}^{T}+B^{T}P\right)x\left(t\right)+M_{1}^{T}w+B^{T}r\left(t\right)\right)\right]+\mathrm{Dw}\left(t\right) \end{array}$$

where $P\in R^{n}$ is symmetric solution of the algebraic Riccati Equation (24) and vector r(t) can be obtained by using backward integration of Equation (25).

(28) $$\begin{array}{cc} r(t) & =\int_{0}^{t_{p}}e^{A_{c}\left(t-\tau \right)}\left({\mathrm{PD}}_{n}-N_{n}\right)w\left(\tau \right)d\tau \end{array}$$

where $A_{c}=A_{n}-{\mathrm{BR}}^{-1}B^{T}P$ is an asymptotically closed loop stable system matrix. The exponential function in Equation (28) will exponentially decrease with time and the knowledge of future disturbance will become irrelevant to the system performance. Furthermore, for $\tau >t+t_{p},w\left(t\right)$ is not available.

## 5. Simulation Results and Discussion

**Figure 3:**
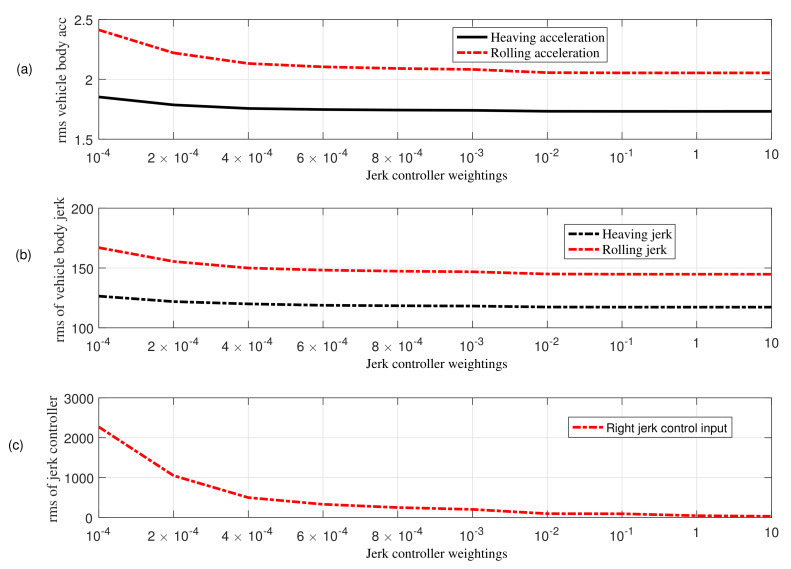
Comparison based on rms values obtained using different weights in the presence of bump velocity input; (**a**) vehicle body acceleration (**b**) vehicle body jerk (**c**) jerk of active aerodynamic surfaces.

**Figure 4:**
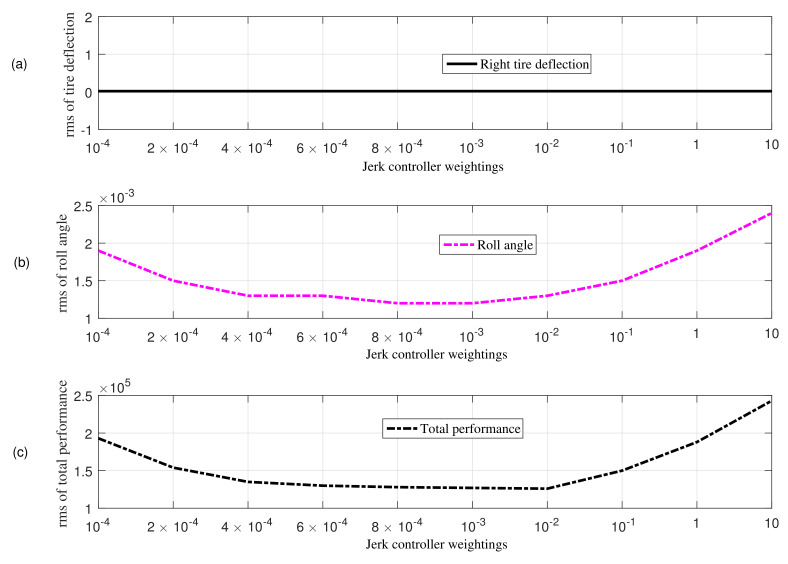
Comparison based on rms values obtained using different weights in the presence of bump velocity input; (**a**) tire deflection (**b**) roll angle (**c**) total performance.

In this section, simulation results for a four-degree-of-freedom half-car model with active aerodynamic surfaces traveling at 20 m/s are carried out using MATLAB. The results are discussed using various weighting factors for jerk control inputs. Table 2 shows weighting factors used in the performance index in Equation (13). Figure 3 and Figure 4 collects rms values for each individual performance of heaving and rolling jerk, heaving and rolling acceleration, tire and suspension deflection, jerk control inputs, and total vehicle performance. Figure 4 shows that the vehicle’s entire performance is best at $10^{-2}$, hence can be suitable choice to manage the control jerk of AAS to enhance ride comfort without degrading other performance parameters. Therefore, considering the aim of this paper, the weighting parameter $\rho_{7}$ for the proposed anti-jerk preview controller is set to $10^{-2}$, whereas $\rho_{7}$ for optimal preview control (OPC) strategy without jerk is set to $10^{-4}$.

The efficiency of the proposed anti-jerk preview control approach is evaluated via two types of characteristics. In the first type, the proposed AJPC and OPC strategy without jerk are compared to evaluate simulation results for frequency domain characteristics. In the frequency domain, opposite-phase roll inputs are evaluated, and simulation results show that, when compared to the OPC strategy without jerk, the proposed AJPC approach successfully enhances vehicle performance. The second type of characteristics is time domain characteristics, which are discussed in two separate scenarios for a bump input and an asphalt road, respectively. Comparing AJPC and OPC strategy without jerk, the results indicate that the proposed control strategy successfully enhances the performance of active aerodynamic surfaces, allowing the AAS to run more smoothly to efficiently manage the control jerk, and reduces the vehicle body jerk to enhance ride comfort.

### 5.1. Frequency Domain Characteristics

Frequency domain characteristics are one of the most significant characteristics of a vehicle suspension control system to evaluate its performance in the presence of various disturbances. In frequency domain analysis the amplitude ratio between the steady state output and disturbance input is described by the magnitude of the frequency response function. Frequency response provides a great deal of information about just how your system responds to disturbances in different frequency bands. In this paper, the half-car model, which can be excited by two kind excitation inputs i.e., heaving input or rolling input to investigate the performance of the proposed AJPC strategy. Therefore, a harmonic disturbance with appropriate phasing is used to induce roll motion to acquire a better understanding of the performance of closed loop systems in the frequency domain. For AJPC and OPC strategy without jerk, the frequency domain properties of roll acceleration, suspension, and tyre deflection are compared.

The frequency domain characteristics are determined using Fourier transform of the closed loop system and Equation (28) as:

(29) $$\begin{array}{cc} \frac{X(j\omega )}{w(j\omega )} & ={\left(j\omega I-A_{c}\right)}^{-1}\left(D_{n}-BR^{-1}B^{T}\int_{0}^{t_{p}}e^{A_{c}\sigma }\left({\mathrm{PD}}_{n}.-N_{n}\right)e^{-j\omega \sigma }d\sigma \right){\left[e^{-j\omega \sigma_{1}},e^{-j\omega \sigma_{2}}\right]}^{T} \end{array}$$

**Figure 5:**
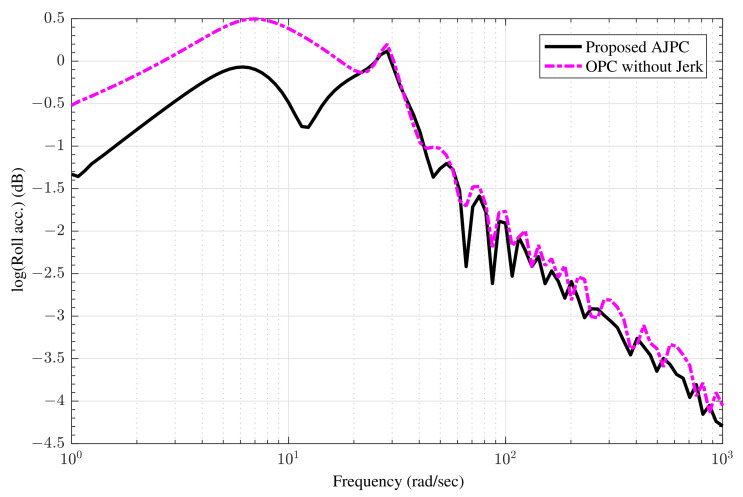
Frequency response comparison of rolling acceleration between proposed AJPC strategy and OPC strategy without jerk.

**Figure 6:**
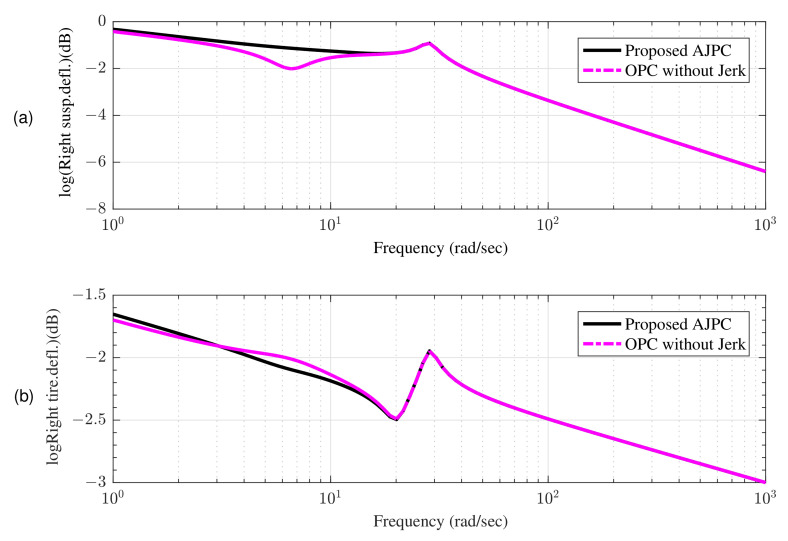
Frequency response to a rolling input using proposed AJPC strategy and OPC strategy without jerk; (**a**) right suspension deflection (**b**) right tire deflection.

The amplitude ratio between body motions and disturbance inputs can be determined using the aforementioned equations without taking body forces into account. To derive frequency domain characteristics, only roll inputs that are opposite in phase as $e^{-j\omega 0}=1,$$e^{-j\omega \pi }=-1$ are considered. The influence of the proposed AJPC strategy on ride comfort and road holding capability can be seen in the frequency domain characteristics shown in Figure 5 and Figure 6. Figure 5 shows that compared to OPC strategy without jerk, the roll acceleration by the proposed AJPC is reduced, which indicates an improvement in ride comfort. The results for the right suspension and tire deflections are shown in Figure 6a,b, which shows that both tire and suspension deflections for the proposed AJPC strategy and OPC without jerk are same. Hence, the simulation results for the frequency domain characteristics show that the proposed AJPC strategy enhances ride comfort without compromising road holding capability of the vehicle. However, the main objectives of this paper are to effectively manage the control jerk to enhance the ride quality of the vehicle. These objectives can be discussed in detail using time domain characteristics.

### 5.2. Time Domain Characteristics

In this section, the simulation results are discussed for time domain characteristics, which are very insightful for the transient as well steady-state response of the system. Therefore, the simulation results are carried out for the jerk of active aerodynamic surfaces, vehicle body jerk, vehicle body acceleration, tyre deflection, suspension deflection, and roll angle. The results demonstrate that in the presence of disturbances, the proposed AJPC strategy assists the vehicle better than the OPC strategy without jerk in achieving optimum control jerk, reducing vehicle body jerk and acceleration to improve the ride comfort, and tire deflection to maintain the road holding capability.

The performance of vehicle is evaluated for two different scenarios: a bump velocity input and an asphalt velocity road disturbances.

**Figure 7:**
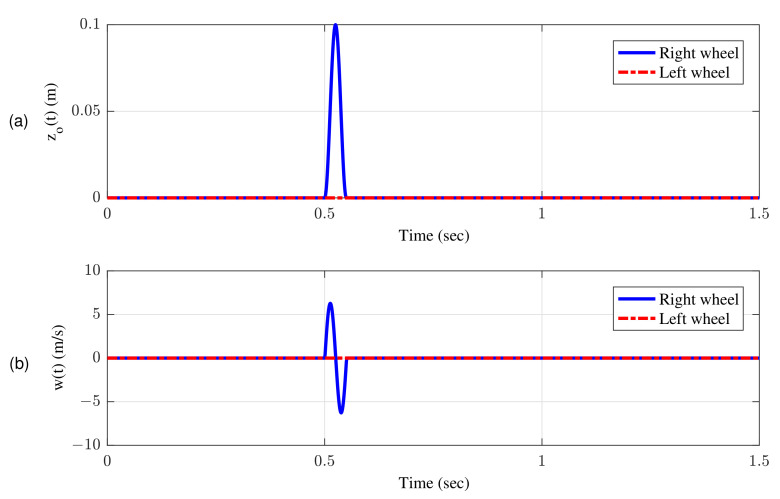
Road disturbance; (**a**) bump position input (**b**) bump velocity input.

**Figure 8:**
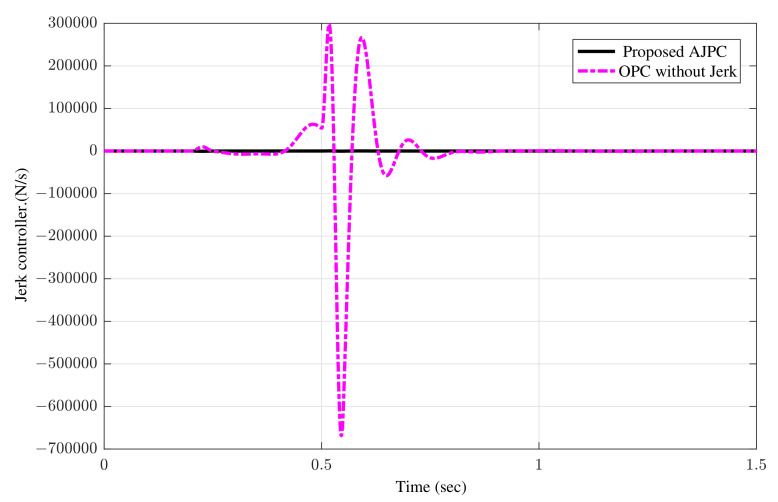
Time domain response of control jerk to a bump velocity input using proposed AJPC strategy and OPC strategy without jerk.

**Figure 9:**
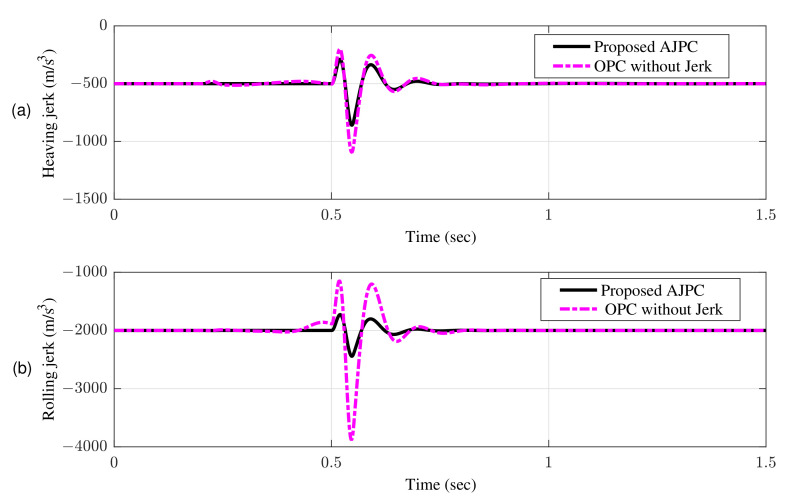
Time domain response of vehicle body jerk to a bump velocity input using proposed AJPC strategy and OPC strategy without jerk; (**a**) heaving jerk (**b**) rolling jerk.

**Figure 10:**
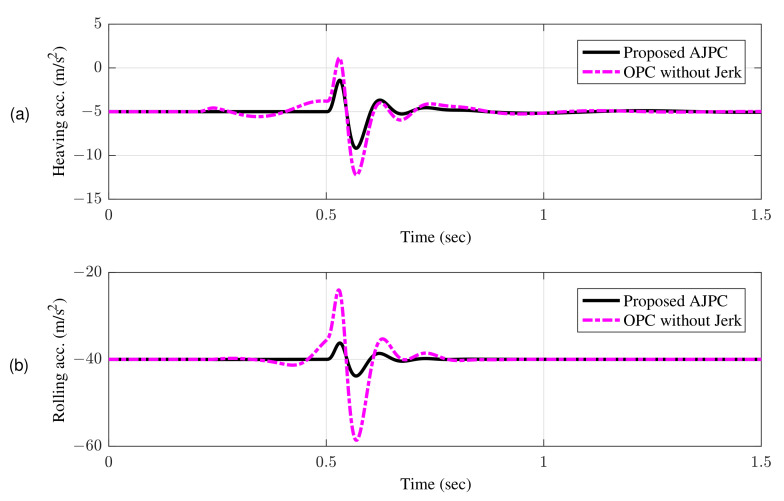
Time domain response of vehicle body acceleration to a bump velocity input using proposed AJPC strategy and OPC strategy without jerk; (**a**) heaving acceleration (**b**) rolling acceleration.

Figure 7a shows bump input and its associated velocity input shown in Figure 7b given to the right wheel. Figure 8 shows simulation results for the jerk of AAS considering proposed AJPC and OPC strategy without jerk, which indicates that compared to the OPC strategy without jerk the proposed AJPC strategy successfully reduces the jerk of AAS. As a result the AAS ovoids any abrupt moment, which has a direct impact on ride comfort. Figure 9a,b shows the simulation results for heaving and rolling jerk, respectively, which illustrate that, when compared to the OPC strategy without jerks, the proposed AJPC successfully reduces both heaving and rolling jerks, indicating an improvement in ride comfort. Figure 10a,b shows the simulation results for heaving and rolling accelerations, respectively. When comparing the AJPC and OPC strategy without jerk, it indicates that the heaving and rolling acceleration for the proposed AJPC is lower than that of OPC strategy.

**Figure 11:**
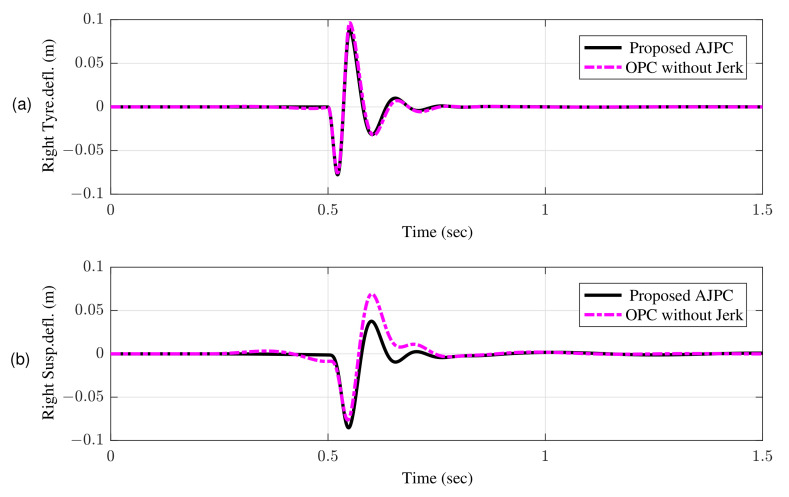
Time domain response of tire and suspension deflection to a bump velocity input using proposed AJPC strategy and OPC strategy without jerk; (**a**) right tire deflection (**b**) right suspension deflection.

This guarantees that aerodynamic surfaces operate smoothly and assist the vehicle in enhancing ride comfort and road holding capability. Figure 11a,b shows the simulation results for the right tire and right suspension deflections, respectively, which shows that proposed control strategy do not let the vehicle to weaken the tire’s grip on the road.

**Figure 12:**
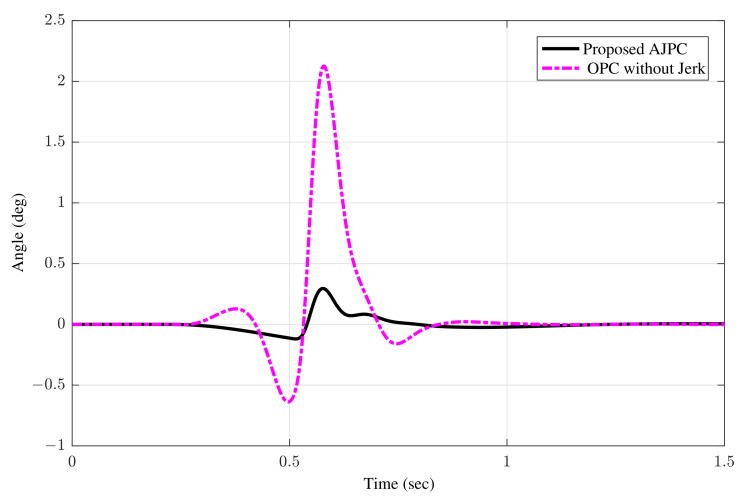
Time domain response of roll angle to a bump velocity input using proposed AJPC strategy and OPC strategy without jerk.

The simulation results for suspension deflection shows that for the proposed AJPC strategy, suspension deflection is within the allowable space 0.08 m range, as defined by [48]. Moreover, the suspension deflection for OPC is more than the allowable space. Figure 12 show the simulation results for the roll angle using the proposed AJPC and the OPC strategy without jerk, respectively. When comparing, the results demonstrate that the designed AJPC has a substantially lower roll angle than OPC strategy without jerk.

The simulation results for an asphalt road disturbance defined in (12) and its corresponding velocity disturbance are illustrated. Table 3 shows the comparison of performance parameters of vehicle systems.

**Figure 13:**
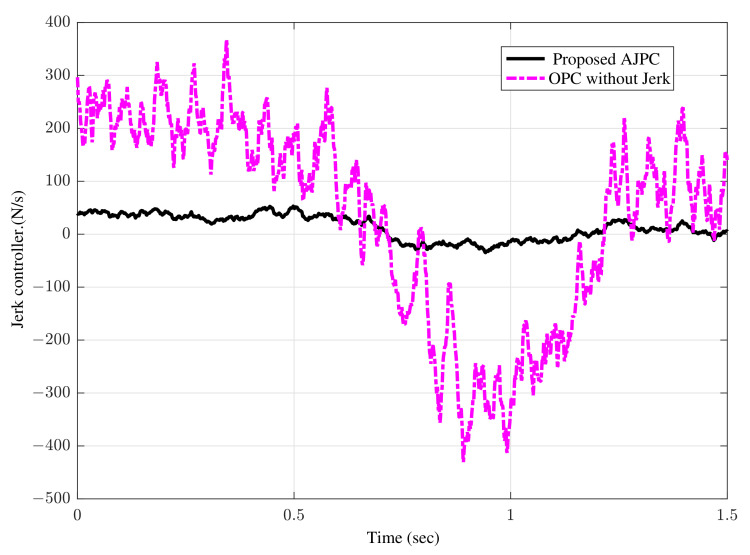
Time domain response of control jerk to an asphalt road using proposed AJPC strategy and OPC strategy without jerk.

**Figure 14:**
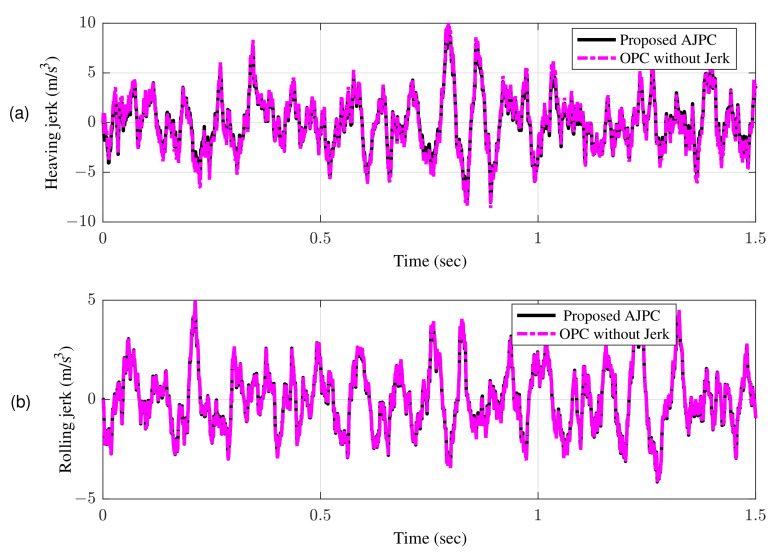
Time domain response of vehicle body jerk to an asphal road using proposed AJPC strategy and OPC strategy without jerk; (**a**) heaving jerk (**b**) rolling jerk.

**Figure 15:**
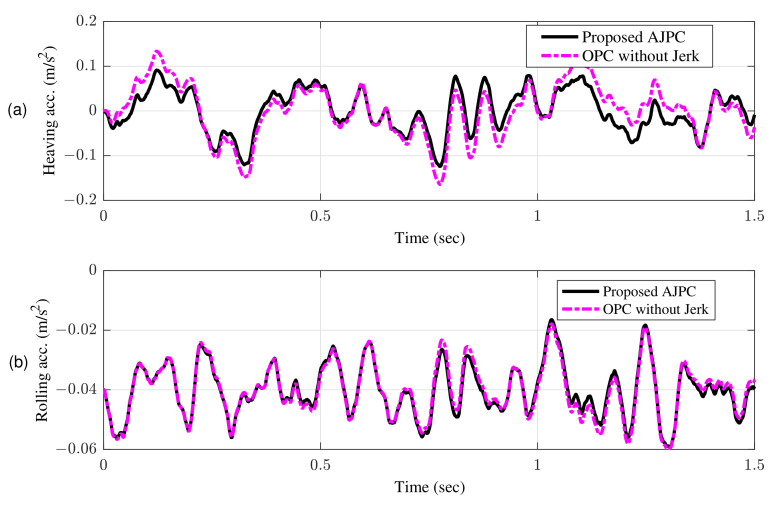
Time domain response of vehicle body acceleration to an asphalt road using proposed AJPC strategy and OPC strategy without jerk; (**a**) heaving acceleration (**b**) rolling acceleration.

The simulation results for the jerk control inputs AJPC and OPC strategy without jerk are shown in Figure 13, respectively. Comparing the results in Figure 13 indicates that the performance of AAS for the AJPC is significantly smoother with a lower jerk than for the OPC strategy without jerk, confirming the purpose of the proposed anti-jerk optimal preview control technique. Figure 14a,b shows the simulation results for heaving and rolling jerks, respectively. The results show that in case of AJPC the heaving jerk is 8% reduced, while the rolling jerk is 3% reduced. The simulation results for heaving and rolling acceleration are shown in Figure 15, which shows that the proposed AJPC successfully reduces both heaving and rolling acceleration when compared to the OPC strategy without jerk.

**Figure 16:**
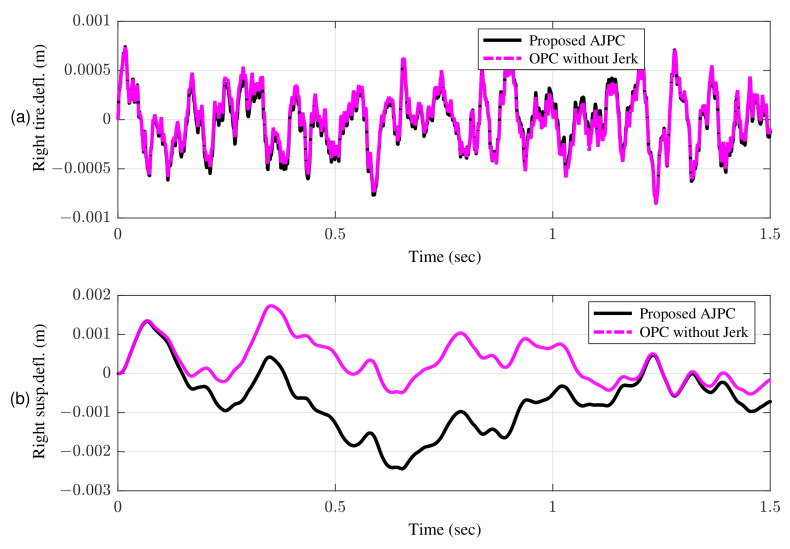
Time domain comparison of tire and suspension deflection between proposed AJPC strategy and OPC strategy without jerk; (**a**) right tire deflection (**b**) right suspension deflection.

AJPC results in 14% decrease in heaving acceleration and a 2% decrease in rolling acceleration. Hence, by reducing both jerk and acceleration in attitude motion of the vehicle body, the proposed AJPC strategy successfully enhances the ride comfort. The rms values in Table 3 shows that compared to OPC strategy without jerk, AJPC has an 4% lower tire deflection. Figure 16 shows the results for suspension deflection which shows that compared to OPC the proposed AJPC has lower suspension deflection. As a result, the capacity to hold the road has improved.

**Remark** **1.** *The challenge of how to put the recommended strategy into practice arises since it is preferable to utilize a control force due to the availability of the force actuator. However, a discrete time formulation of their derivative using the change in actuator force Δu over discrete time periods is also possible. The advantage of discrete time implementation is that if the computer fails, the output force remains unchanged. Instead of sustaining an undesired force, it will keep the previous force value.*

## 6. Conclusions

In this paper, an anti-jerk preview control scheme is designed for a half car equipped with active aerodynamic surfaces to improve the performance of AAS and enhance ride comfort and road holding capability of the vehicle. The proposed control approach consists of feed-forward part using future road disturbances and feedback part to deal with the tracking error. To validate the performance of the suggested approach, the vehicle is subjected to two types of road disturbances a bump input and an asphalt road. The efficient performance of the proposed control strategy is analyzed by frequency and time domain characteristics. Frequency domain characteristics are discussed for the ride comfort and road holding capability only, while the time domain characteristics are discussed for the performance of AAS, ride comfort, and road holding.The main objective of this work is to avoid abrupt moment of AAS, which has a direct impact on the ride comfort. The simulation results show that the proposed AJPC strategy has successfully reduced the control jerk to improve the performance of AAS, and reduced heaving and rolling jerk, heaving and rolling acceleration to enhance ride comfort without degrading road holding capability. The simulation results are displayed in MATLAB, demonstrating the superiority of the proposed AJPC scheme over the OPC strategy without jerk.

The current research work can be envisaged in the near future to design the proposed control strategy by considering the following recommendations.

- The proposed control strategy can be investigated considering the actual model of active aerodynamic surfaces with experimental implementations or with some commercial software such as CarSim or CarMaker.
- More advanced robust, and intelligent control algorithms can be considered in the future to tackle both road as well as air disturbances.

## Figures and Tables

**Table 1:** Physical parameters of half-car model.

Symbol	Description	Value	Unit
*M*	Vehicle body mass	500	Kg
*I*	Moment of inertia	274	Kg · m${}^{2}$
$m_{1},m_{2}$	Vehicle unsprung mass	25	Kg
$k_{s_{1}},k_{s_{2}}$	Suspension stiffness	10	kN/m
$k_{t_{1}},\;k_{t_{2}}$	Tire stiffness	1	kN/m
$b_{s_{1}},b_{s_{2}}$	Damping coefficients	1	kN/m
*a*	Distance of C.M from right side	0.74	m
*b*	Distance of C.M from left side	0.74	m
*h*	Height of C.M from the ground	0.70	m

**Table 2:** Two types weighting factors used in performance indices.

Weighting Constants	Targets	AJPC	OPC
$\rho_{1}$	Heaving acceleration	1	1
$\rho_{2}$	Rolling acceleration	1	1
$\rho_{3}$	Suspension deflection	$10^{4}$	$10^{4}$
$\rho_{5}$	Tire deflection	$10^{6}$	$10^{6}$
$\rho_{7}$	Jerk controller	$10^{-2}$	$10^{-4}$

**Table 3:** Root mean square error (RMSE) values of performance parameters of half car subjected to an asphalt road.

Performance Parameters	OPC	AJPC
Right jerk control input	100	15
Heaving jerk	100	92.88
Roll jerk	100	97.6
Heaving acceleration	100	86
Rolling acceleration	100	98
Right tyre deflection	100	96
Right suspension deflection	100	89
Roll angle	100	96
Total performance	100	96

## Appendix A

For the state-space model given in (14) the non-zero entries for the system matrix A are given as:

$$\begin{array}{ccc} & a_{12}=1,a_{23}=1,a_{31}=\frac{m_{2}\cdot \left(b_{s_{1}}+k_{s_{1}}\right)+m_{1}\cdot \left(b_{s_{2}}+k_{s_{2}}\right)}{M}, & \\ & a_{32}=\frac{m_{2}\cdot b_{s_{1}}^{2}-m_{1}\left(\cdot b_{s_{2}}^{2}-m_{2}\cdot \left(k_{s_{1}}+k_{s_{2}}\right)\right)}{M}, & \\ & a_{33}=-\frac{b_{s_{1}}+b_{s_{2}}}{M},a_{34}=\frac{m_{2}\cdot ab_{s_{1}}\cdot k_{s_{1}}-m_{1}\cdot bb_{s_{2}}\cdot k_{s_{2}}\cdot }{M}, & \\ & a_{35}=\frac{m_{2}\cdot a\cdot b_{s_{1}}^{2}-m_{1}\left(\cdot b\cdot b_{s_{2}}^{2}-m_{2}\cdot \left(b\cdot k_{s_{2}}-a\cdot k_{s_{1}}\right)\right)}{M} & \\ & a_{36}=\frac{b_{s_{2}}\cdot b-a\cdot b_{s_{1}}}{M},a_{37}=-\frac{b_{s_{1}}\cdot k_{t_{1}}}{M\cdot m_{1}},a_{38}=\frac{m_{1}\cdot k_{s_{1}}-b_{s_{1}}^{2}}{M}, & \\ & a_{39}=\frac{b_{s_{2}}\cdot k_{t_{2}}}{M\cdot m_{2}},a_{310}=\frac{m_{2}\cdot k_{s_{2}}-b_{s_{2}}^{2}}{M},a_{311}=-\frac{b_{s_{1}}\cdot k_{s_{1}}}{M\cdot m_{1}}, & \\ & a_{312}=-\frac{b_{s_{2}}\cdot k_{s_{2}}}{M\cdot m_{2}},a_{45}=a_{56}=1, & \\ & a_{61}=\frac{m_{2}\cdot a\cdot b_{s_{1}}\cdot k_{s_{1}}-m_{1}\cdot b\cdot b_{s_{2}}\cdot k_{s_{2}}}{I}, & \\ & a_{62}=\frac{k_{s_{2}}\cdot b-k_{s_{1}}\cdot a}{I}+\frac{m_{2}\cdot a\cdot b_{s_{1}}^{2}-m_{1}\cdot b\cdot b_{s_{2}}^{2}}{I}, & \\ & a_{63}=\frac{b_{s_{2}}\cdot b-b_{s_{1}}\cdot a}{I},a_{64}=\frac{m_{2}\cdot a^{2}\cdot b_{s_{1}}k_{s_{1}}+m_{1}\cdot b^{2}\cdot b_{s_{2}}k_{s_{2}}}{I}, & \\ & a_{65}=\frac{m_{2}\cdot a^{2}\cdot b_{s_{1}}^{2}+m_{1}\cdot b^{2}\cdot b_{s_{2}}^{2}-k_{s_{1}}\cdot a^{2}-k_{s_{2}}\cdot b^{2}}{I}, & \\ & a_{66}=-\frac{b^{2}\cdot b_{s_{2}}+a^{2}\cdot b_{s_{1}}}{I},a_{67}=-\frac{a\cdot b_{s_{1}}\cdot k_{t_{1}}}{I\cdot m_{1}}, & \\ & a_{68}=\frac{m_{1}\cdot a\cdot k_{s_{1}}-a\cdot b_{s_{1}}^{2}}{I},a_{69}=\frac{b\cdot b_{s_{2}}k_{t_{2}}}{I\cdot m_{2}}, & \\ & a_{610}=\frac{m_{2}\cdot b\cdot k_{s_{2}}+b\cdot b_{s_{2}}^{2}}{I},a_{611}=-\frac{a\cdot b_{s_{1}}\cdot k_{s_{1}}}{I\cdot m_{1}}, & \\ & a_{612}=-\frac{b\cdot b_{s_{2}}\cdot k_{s_{2}}}{I\cdot m_{2}},a_{78}=1,a_{81}=\frac{k_{s_{1}}}{m_{1}},a_{82}=\frac{b_{s_{1}}}{m_{1}}, & \\ & a_{84}=\frac{a\cdot k_{s_{1}}}{m_{1}},a_{85}=\frac{a\cdot b_{s_{1}}}{m_{1}},a_{87}=-\frac{k_{t_{1}}}{m_{1}},a_{88}=-\frac{b_{s_{1}}}{m_{1}} & \\ & a_{811}=-\frac{k_{s_{1}}}{m_{1}},a_{910}=1,a_{101}=\frac{k_{s_{2}}}{m_{2}},a_{102}=\frac{b_{s_{2}}}{m_{2}}, & \\ & a_{104}=\frac{b\cdot k_{s_{2}}}{m_{2}},a_{105}=\frac{b\cdot b_{s_{2}}}{m_{2}},a_{109}=-\frac{k_{t_{2}}}{m_{2}},a_{1010}=-\frac{b_{s_{2}}}{m_{2}}, & \\ & a_{118}=a_{1210}=1 \end{array}$$

similarly the non-zero entries of matrix B are

$$\begin{array}{cc} & b_{31}=b_{32}=\frac{1}{m},b_{61}=\frac{a}{I},b_{62}=-\frac{b}{I} \end{array}$$

and the non-zero entries for matrix D are

$$\begin{array}{cc} & d_{71}=d_{92}=-1 \end{array}$$

The entries of weight matrix $Q,N_{1},N_{2},M_{1}$ and $M_{2}$ defined in (12) are given as follows

$$\begin{array}{c} q_{ij}=\left\{\begin{array}{cc} \rho_{1}a_{3i}a_{3j}+\rho_{2}a_{6i}a_{6j} & \;\mathrm{if}i\neq j\\ \psi_{ij} & \;\mathrm{if}i=j \end{array}\right. \end{array}$$

$$\begin{array}{ccc} & n_{1ij}=\rho_{1}a_{3i}b_{3j}+\rho_{2}a_{6i}b_{6j}\;i=1,..,12,\;j=1,2 & \\ & n_{2ij}=\rho_{1}a_{3i}d_{3j}+\rho_{2}a_{6i}d_{6j}\;i=1,..,12,\;j=1,2 & \\ & m_{1ij}=\rho_{1}d_{7i}b_{3j}+\rho_{2}d_{9i}b_{6j}\;i=1,\;j=1,2 & \\ & m_{2ij}=\rho_{1}d_{7i}b_{3j}+\rho_{2}d_{9i}b_{6j}\;i,j=1,2 \end{array}$$

$$\begin{array}{ccc} & \psi_{11}=\rho_{1}a_{31}a_{31}+\rho_{2}a_{61}a_{61}+\rho_{3}, & \\ & \psi_{33}=\rho_{1}a_{33}a_{33}+\rho_{2}a_{63}a_{63}+\rho_{1}, & \\ & \psi_{44}=\rho_{1}a_{34}a_{34}+\rho_{2}a_{64}a_{64}+a\rho_{3}-b\rho_{3}+\rho_{4}, & \\ & \psi_{66}=\rho_{1}a_{66}a_{66}+\rho_{2}a_{66}a_{66}+\rho_{2}, & \\ & \psi_{77}=\rho_{1}a_{37}a_{37}+\rho_{2}a_{67}a_{67}+\rho_{5}, & \\ & \psi_{99}=\rho_{1}a_{39}a_{39}+\rho_{2}a_{69}a_{69}+\rho_{5}, & \\ & \psi_{1010}=\rho_{1}a_{310}a_{310}+\rho_{2}a_{610}a_{610}, & \\ & \psi_{1111}=\rho_{1}a_{311}a_{311}+\rho_{2}a_{611}a_{611}+\rho_{3}, & \\ & \psi_{1212}=\rho_{1}a_{312}a_{312}+\rho_{2}a_{612}a_{612}+\rho_{3} \end{array}$$
